# Vaginal examinations and mistreatment of women during facility-based childbirth in health facilities: secondary analysis of labour observations in Ghana, Guinea and Nigeria

**DOI:** 10.1136/bmjgh-2021-006640

**Published:** 2021-11-17

**Authors:** Kwame Adu-Bonsaffoh, Hedieh Mehrtash, Chris Guure, Ernest Maya, Joshua P Vogel, Theresa Azonima Irinyenikan, Adeniyi Kolade Aderoba, Mamadou Dioulde Balde, Richard Adanu, Meghan A Bohren, Özge Tuncalp

**Affiliations:** 1 Department of Obstetrics Gynaecology, University of Ghana Medical School, Accra, Ghana; 2 Department of Obstetrics and Gynaecology, Korle Bu Teaching Hospital, Accra, Ghana; 3 Department of Sexual and Reproductive Health and Research, including UNDP/UNFPA/UNICEF/WHO/World Bank Special Programme of Research, Development and Research Training in Human Reproduction (HRP), WHO, Geneva, Switzerland; 4 Department of Global Health, University of Washington School of Public Health, Seattle, Washington, USA; 5 Department of Biostatistics, University of Ghana School of Public Health, Accra, Ghana; 6 Department of Population, Family and Reproductive Health, University of Ghana School of Public Health, Accra, Ghana; 7 Maternal, Child, and Adolescent Health Programme, Burnet Institute, Melbourne, Victoria, Australia; 8 Department of Obstetrics and Gynaecology, Faculty of Clinical Sciences, University of Medical Sciences, Ondo City, Nigeria; 9 Department of Obstetrics and Gynaecology, University of Medical Sciences Teaching Hospital, Akure, Nigeria; 10 Department of Obstetrics and Gynaecology, Mother and Child Hospital, Akure, Nigeria; 11 Cellulle de Recherche en Sante de la Reproduction en Guinee (CERREGUI), Conakry, Guinea; 12 University of Ghana School of Public Health, Accra, Ghana; 13 Centre for Health Equity, University of Melbourne School of Population and Global Health, Melbourne, Victoria, Australia; 14 Reproductive Health and Research, WHO, Geneva, Switzerland

**Keywords:** maternal health, obstetrics

## Abstract

**Background:**

Previous research on mistreatment of women during childbirth has focused on physical and verbal abuse, neglect and stigmatisation. However, other manifestations of mistreatment, such as during vaginal examinations, are relatively underexplored. This study explores four types of mistreatment of women during vaginal examinations: (1) non-consented care, (2) sharing of private information, (3) exposure of genitalia and (4) exposure of breasts.

**Methods:**

A secondary analysis of data from the WHO multicountry study ‘How Women Are Treated During Childbirth’ was conducted. The study used direct, continuous labour observations of women giving birth in facilities in Ghana, Guinea and Nigeria. Descriptive and multivariable logistic regression analyses were used to describe the different types of mistreatment of women during vaginal examinations and associated privacy measures (ie, availability of curtains).

**Results:**

Of the 2016 women observed, 1430 (70.9%) underwent any vaginal examination. Across all vaginal examinations, 842/1430 (58.9%) women were observed to receive non-consented care; 233/1430 (16.4%) women had their private information shared; 397/1430 (27.8%) women had their genitalia exposed; and 356/1430 (24.9%) had their breasts exposed. The observed prevalence of mistreatment during vaginal examinations varied across countries. There were country-level differences in the association between absence of privacy measures and mistreatment. Absence of privacy measures was associated with sharing of private information (Ghana: adjusted OR (AOR) 3.8, 95% CI 1.6 to 8.9; Nigeria: AOR 4.9, 95% CI 1.9 to 12.7), genitalia exposure (Ghana: AOR 6.7, 95% CI 2.9 to 14.9; Nigeria: AOR 6.5, 95% CI 2.9 to 14.5), breast exposure (Ghana: AOR 5.9, 95% CI 2.8 to 12.9; Nigeria: AOR 2.7, 95% CI 1.3 to 5.9) and non-consented vaginal examination (Ghana: AOR 2.5, 95% CI 1.4 to 4.7; Guinea: AOR 0.21, 95% CI 0.12 to 0.38).

**Conclusion:**

Our results highlight the need to ensure better communication and consent processes for vaginal examination during childbirth. In some settings, measures such as availability of curtains were helpful to reduce women’s exposure and sharing of private information, but context-specific interventions will be required to achieve respectful maternity care globally.

**Key questions**

**What is already known?**

- Most studies on mistreatment of women during childbirth have reported measures related to physical abuse, verbal abuse, neglect, and stigma and discrimination.
- There is limited and varied evidence using empirical data to explore manifestations of different types of mistreatment during the vaginal examinations, which are a particularly sensitive time for women giving birth.

**What are the new findings?**

- Using a standardised labour observation tool across three countries, we provide evidence that women are experiencing different forms of mistreatment across any vaginal examinations, including (1) non-consented care, (2) sharing of private information, (3) exposure of genitalia and (4) exposure of breasts.
- Non-consented care was common across all countries during vaginal examinations; we also found that the other forms of mistreatment (sharing of private information and exposure of genitalia breasts) were associated with an absence of privacy measures such as availability of curtains, and varied depending on the country.

**What do the new findings imply?**

- Across all vaginal examinations and countries, three out of five women were observed to receive non-consented care.
- Our study shows the importance of how privacy measures can be important in promoting respectful maternity care, particularly women’s rights to privacy and confidentiality.
- At the interpersonal level, education and counselling on vaginal examinations as part of routine antenatal care may help women prepare for what to expect during childbirth.
- At the health system level, enabling environments to support healthcare workers, including appropriate continuing education/training, supervision and supportive policies, is needed to promote respectful care.

## Background

Women’s experiences of mistreatment during childbirth in health facilities is a major public health issue globally.^1–3^ The WHO declared that ‘every woman has the right to the highest attainable standard of health, which includes the right to dignified, respectful healthcare’.^4^ This statement emerged following growing evidence that mistreatment of women during childbirth is associated with unacceptable short-term and long-term adverse effects on maternal health, including future reluctance to seek facility-based care and violations of women’s rights to care.^5–7^ Moreover, the 2018 WHO intrapartum care guideline recommends respectful maternity care for all women, including effective communication, companionship during labour and birth, maintenance of privacy and confidentiality, freedom from harm and mistreatment, and informed choice.^3^

Most studies on mistreatment of women have reported measures related to verbal abuse, physical abuse, neglect, stigma and discrimination.^8–10^ However, other manifestations of mistreatment, such as during vaginal examinations, are relatively underexplored. Previous studies have reported that women experience psychologically traumatic vaginal examinations, although the extent of its occurrence and effect on childbirth has not been extensively studied.^11 12^ In a recent WHO study, more than half of women reported an uncomfortable experience of vaginal examination while being admitted for childbirth, with about 60% not being informed or consenting to being examined.^13^ Negative experiences during vaginal examinations have likewise been reported in South Africa and Tanzania.^14 15^ Mistreatment during vaginal examination is not limited to low-income and middle-income countries.^1^ For instance, de Klerk *et al* reported women’s experiences of painful, insensitive and disrespectful vaginal examinations by healthcare professionals in the Netherlands.^16^

Vaginal examination during labour is an important clinical assessment used to determine the progress of labour by assessing cervical dilation and relevant fetal parameters. WHO recommends that in healthy low-risk women, vaginal examination should be performed at intervals of every 4 hours for routine assessment of active first stage of labour, with the recommendation remarks specifying that priority should be given to restricting the frequency and total number of examinations, as well as limiting the number of caregivers conducting the examinations.^17^ Given the inherently sensitive and invasive nature of vaginal examinations, it is critical to obtain informed consent and permission from the woman prior to conducting the examination and to communicate clearly the findings of the examination to her using a language that she understands.

Despite their usefulness in assessing progression of labour, vaginal examinations can be viewed or experienced negatively by women.^11 17 18^ This may be particularly true for women giving birth for the first time or women who may not have benefited from clear communication about when and why vaginal examinations are needed. This can result in women feeling powerless or experiencing severe pain and discomfort.^11^ For instance, a study in Palestine reported that women undergoing vaginal examinations felt they were treated insensitively by health professionals and that the process lacked privacy, respect and dignity.^19^ Similarly, a recent study in Kenya found that inappropriate professional standards of care were characterised by lack of informed consent prior to vaginal examinations, frequent examinations and lack of privacy during examinations.^20^ These negative experiences of vaginal examinations can result in women feeling embarrassed, physically traumatised, without communication, and lacking trust, dignity and respect.^21^ More recently, similar sentiments and experiences have been reported in other countries including the Netherlands,^16^ Iran^22^ and Turkey.^23^

The objective of this study was to assess the determinants of mistreatment of women during vaginal examination in Ghana, Guinea and Nigeria using data collected through direct continuous observation of women during facility-based childbirth.

## Methods

### Study design and participation

This study is a secondary analysis of data that were collected for a larger cross-sectional study designed to develop and validate two tools (community survey and labour observation tools) to measure the mistreatment of women during childbirth in health facilities. The protocols for the formative phase and methodological development of these tools are available,^24 25^ and the methods and results of the primary analysis have been published.^13^ Briefly, in each country, three facilities were purposively selected based on the following inclusion criteria^25^: (1) facilities not included in the formative phase of developing these tools; (2) secondary-level facility or higher; (3) ≥200 births per month; and (4) well-defined community catchment area. This analysis used data collected from the labour observations, which were conducted in Ghana, Guinea and Nigeria.

### Data collection and management

#### Participants

Women were eligible for labour observations if they were admitted to the participating health facilities for childbirth in early established or active labour (<6 cm cervical dilation), were ≥15 years, willing and able to participate, and provided written informed consent.^13^ All labour observations were continuous, one-to-one observations of women by independent data collectors from admission, throughout labour and childbirth, until 2 hours postpartum. Data collection took place 24 hours/day, 7 days/week. Data were collected using digital, tablet-based tools with built-in quality checks and validation rules (BLU Studio XL2, Android; BLU Products, Miami, Florida, USA). Data were submitted securely to a central database (WHO, Geneva) using a 3G cellular connection or wireless Internet.

#### Measurement tool

The labour observation tool was developed using an iterative mixed-methods approach which is described in detail elsewhere.^24 25^ In short, the tool consisted of (1) an admission form; (2) an incident report form; and (3) a childbirth, interventions and discharge form. The admission form was completed once (immediately after enrolment) for all women and included screening questions and sociodemographic information. The incident report form was completed if, and only if, one of the following events occurred: physical abuse, verbal abuse, stigma and discrimination, or a vaginal examination, and could be completed and submitted multiple times (ie, repeating data collection for multiple events). For instances of vaginal examination, the form captured information about whether the sharing of private information, consent, privacy and confidentiality were observed or not, and were reported as ‘incidents’ because multiple vaginal examinations can occur throughout a woman’s labour.^24^ The childbirth, interventions and discharge forms were completed once at the end of the observation for all women and included pain relief, mobilisation, fluids, companionship, fees, neglect, privacy, health outcomes and interventions.

#### Outcome and predictor variables

In this analysis, the outcome of interest was mistreatment of women during vaginal examination and consisted of four key variables: (1) non-consented vaginal examination (the staff did not provide prior information or obtain permission or both); (2) breast exposure during vaginal examination (vaginal examination was conducted in a way that other patients, visitors or non-medical staff could see the woman’s breasts); (3) genitalia exposure during vaginal examination (vaginal examination was conducted in a way that other patients, visitors, non-medical staff could see the woman’s genitalia); and (4) private information shared during vaginal examination (defined as ‘a woman’s private health information was shared in a way that other non-medical staff, visitors, patients or non-consented family members could hear’). The independent variables of interest were women’s characteristics (sociodemographic and obstetric) and availability of curtains during labour (eg, to maintain privacy, as it was standard practice in the study settings for two or more women to be managed in the same room during labour and birth).

### Data analysis

Descriptive analyses and χ^2^ tests of associations were conducted to assess sociodemographic characteristics, obstetric characteristics and whether women experienced a vaginal examination. We examined the differences between mistreatment of women during vaginal examination across all examinations (n=1430). Additionally, we explored multiple examinations to investigate how mistreatment might differ for subsequent vaginal examinations, by considering women at their first vaginal exam (n=1430), at their second vaginal examination (among women who received at least two examinations (n=839) and at their third vaginal examination (among women who received at least three examinations (n=419) by country (online supplemental annex 1).

10.1136/bmjgh-2021-006640.supp1 Supplementary data

In the primary analysis of these data, we found that women who reported no use of privacy measures (eg, curtains) during labour and childbirth were more likely to report lack of privacy during vaginal examination compared to women with privacy measures.^13^ We therefore hypothesised for this analysis that the absence of privacy measures may be associated with different types of mistreatment during vaginal examinations specifically. As such, multivariable logistic regression models were fitted to evaluate the association between women’s characteristics, availability of curtains during labour and the mistreatment of women during vaginal examination using four variables of interest: non-consented care, genitalia exposure, breast exposure and private information shared. Due to the presence of effect modification by country, models were stratified by country. All models were adjusted for maternal age, education and marital status. Data were analysed using SAS V.9.4.

### Patient and public involvement

A technical consultation with representatives from advocacy groups, non-governmental organisations, research organisations, universities, professional associations, and United Nations agencies was held at the WHO in November 2013, which informed the design of the multicountry study. Women who recently gave birth were involved in content validity testing and providing feedback on the validity testing of the community survey tool, which was harmonised with the labour observation tool for comparability.

## Results

**Figure 1:**
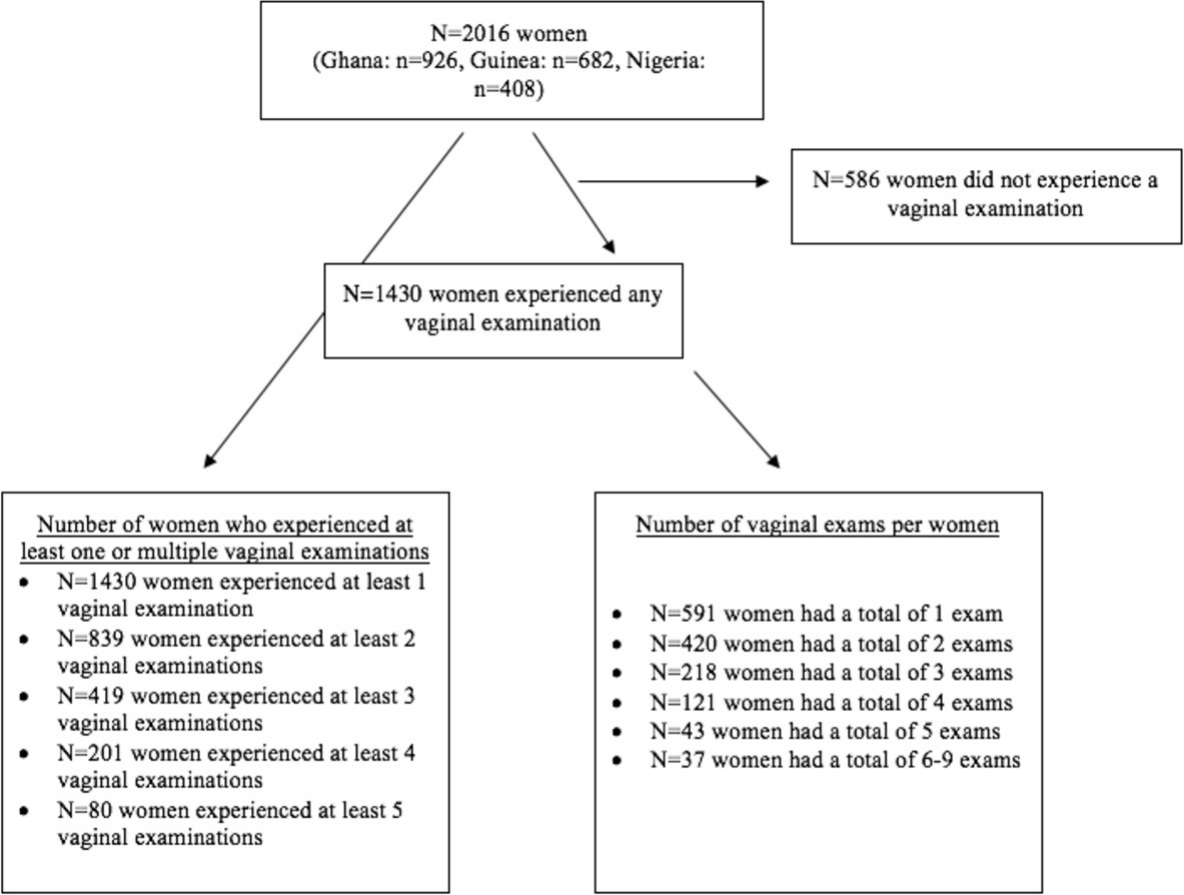
Flow diagram of vaginal examinations across the study sample.

Figure 1 describes the number of vaginal examinations experienced by women. There were 2016 women across the three countries in the study (Ghana: n=926, Guinea: n=682 and Nigeria: n=408). Among the 2016 women, 586 (29.1%) women did not experience any vaginal examination during the observation period. Of the 2016 women, 1430 (70.9%) experienced at least one vaginal examination; 839 (41.6%) experienced at least two examinations; 419 (20.8%) experienced at least three examinations; 201 (9.9%) experienced at least four examinations; and 80 (3.9%) experienced five examinations.

Table 1 shows the sociodemographic and obstetric characteristics of women, stratified by experience of vaginal examination. Overall, women who did not experience vaginal examinations during the labour observation were younger, had no education and had a lower number of previous births. Among women who experienced a vaginal examination, 785/1430 (55.2%) were observed to have an absence of curtains during labour, whereas among women who did not experience a vaginal examination, this was 240/586 (40.9%). The median duration of observation was similar for women who experienced a vaginal examination (5.5 (range 3.9–8.5) hours), and who did not (4.8 (range 3.6–6.8) hours).

**Table 1:** Sociodemographic and obstetric characteristics of women by experience of vaginal examination

	Experienced one or more vaginal examinations (n=1430)	Did not experience a vaginal examination (n=586)	Total (n=2016)
	n (%)	n (%)	n (%)
Maternal age (years)*			
15–19	159 (11.1)	115 (19.6)	274 (13.6)
20–29	705 (49.3)	304 (51.9)	1009 (50.1)
≥30	566 (39.6)	167 (28.5)	733 (36.4)
Marital status			
Single	136 (9.5)	69 (11.8)	205 (10.2)
Currently married/cohabitating	1251 (87.5)	505 (86.2)	1756 (87.1)
Other	43 (3.0)	12 (2.0)	55 (2.7)
Education*			
No education	199 (13.9)	172 (29.4)	371 (18.4)
Some primary	134 (9.4)	80 (13.7)	214 (10.6)
Some secondary	389 (27.2)	128 (21.8)	517 (25.6)
Complete secondary	396 (27.7)	130 (22.2)	526 (26.1)
Complete tertiary	273 (19.1)	57 (9.7)	330 (16.4)
Vocational/other/unknown	39 (2.7)	19 (3.2)	58 (2.9)
Number of previous births (index included)			
1	525 (36.7)	220 (37.5)	745 (36.9)
2	378 (26.4)	138 (23.6)	516 (25.6)
3	244 (17.1)	84 (14.3)	328 (16.3)
4	275 (19.2)	143 (24.4)	418 (20.7)
Unknown	8 (0.6)	1 (0.2)	9 (0.5)
Curtains during labour			
Yes	789 (55.2)	325 (55.5)	1114 (53.3)
No	593 (41.5)	240 (41.0)	833 (41.3)
Unknown	48 (3.4)	21 (3.6)	69 (3.4)
Duration of observation			
Mean (SD) in hours	6.7 (6.1)	5.2 (5.8)	6.3 (6.0)
Median (IQR) in hours	5.5 (3.9–8.5)	4.8 (3.6–6.8)	5.2 (3.8–8.0)
Mode of birth			
Vaginal birth	1189 (83.1)	491 (83.8)	1680 (83.3)
Caesarean birth	190 (13.3)	71 (12.1)	261 (12.9)
Other/unknown	51 (3.6)	24 (4.1)	75 (3.7)
Birth companion*			
Yes	112 (7.8)	14 (2.4)	126 (6.2)
No	1318 (92.2)	572 (97.6)	1890 (93.8)

*P value <0.005.

IQR, Interquartile range; SD, Standard Deviation.

**Figure 2:**
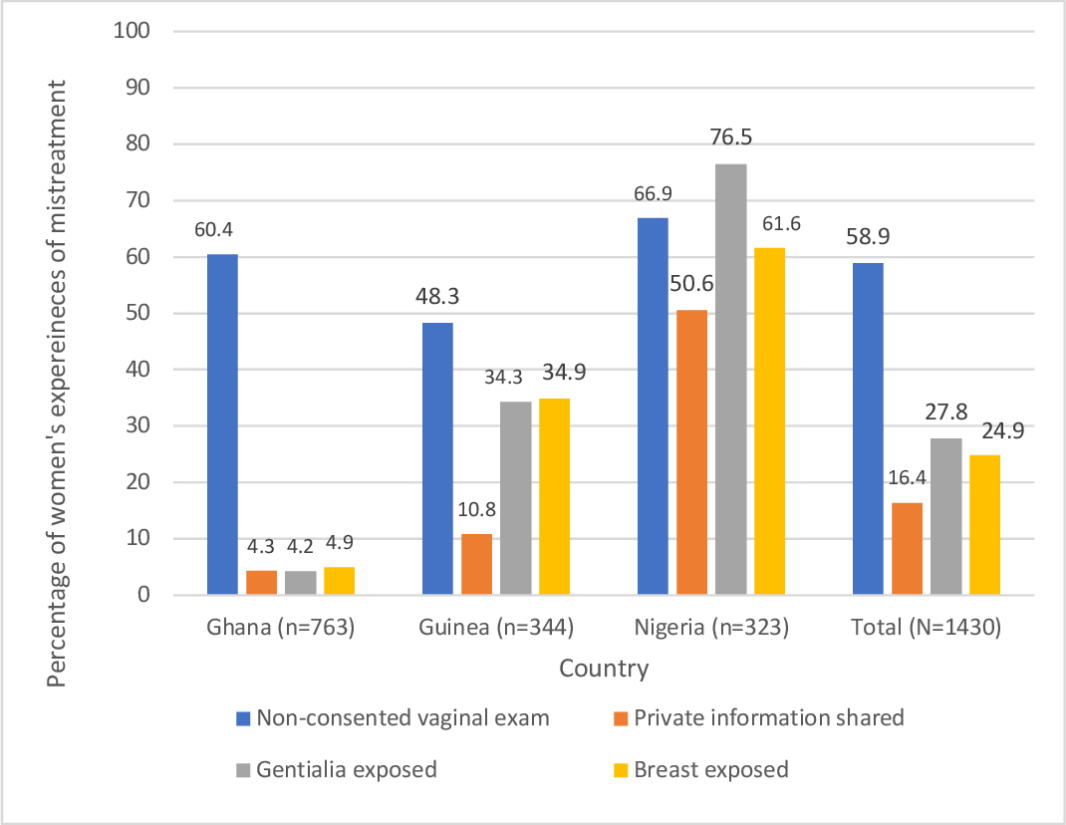
Different types of mistreatment during any vaginal examination by country.

Figure 2 shows the different types of mistreatment across any vaginal examinations. Women’s experience of non-consented care was consistently high across any vaginal examinations. Women’s private information shared during vaginal examinations was highest in Nigeria across any vaginal examinations, compared with similar lower trends in Ghana and Guinea. Similarly, women’s breasts exposed during vaginal examinations was highest in Nigeria across any vaginal examinations, followed by Guinea and Guinea. Women’s genitalia exposed during vaginal examinations was highest in Nigeria across any vaginal examinations, followed by Guinea and Ghana. Online supplemental annex 1 shows that the trends in different types of mistreatment across are similar across multiple vaginal examinations.

### Association between curtains during labour and vaginal examination

Table 2 shows the results of the adjusted multivariable logistic regression model examining whether the absence of curtains is associated with a woman’s experience of mistreatment during any vaginal examination, adjusting for maternal age, education and marital status. Overall, the association between availability of curtains and types of mistreatment during vaginal examination varied by country. The odds of a woman’s genitalia exposure during vaginal examination was six times higher among women without curtains during labour in Ghana and Nigeria (Ghana: AOR 6.7, 95% CI 2.9 to 14.9; Nigeria: AOR 6.5, 95% CI 2.9 to 14.5) in contrast to Guinea (AOR: 0.04, 95% CI 0.02 to 0.08). In Ghana and Nigeria, the absence of curtains during labour was also statistically significantly associated with higher odds of a woman’s breast exposure during vaginal examination (Ghana: AOR 5.9, 95% CI 2.8 to 12.9; Nigeria: AOR 2.7, 96% CI 1.3 to 5.9), whereas in Guinea, it was associated with a lower likelihood (AOR 0.04, 95% CI 0.02 to 0.07). In Ghana and Nigeria, there was also a significant association between the absence of curtains during labour and a woman’s private information shared during vaginal examination (Ghana: AOR 3.8, 95% CI 1.6 to 8.9; Guinea: AOR 1.7, 95% CI 0.7 to 4.2); Nigeria: AOR 4.9, 95% CI 1.9 to 12.7). Also, absence of curtains during labour was associated with increased and reduced odds of non-consented vaginal examination in Ghana (AOR 2.5, 95% CI 1.4 to 4.7) and Guinea (AOR 0.2, 95% CI 0.1 to 0.4), respectively.

**Table 2:** Association between availability of privacy measures and mistreatment during any vaginal examination, by country (N=1430)

	Any non-consented vaginal examination			Any genitalia exposed			Any breast exposed			Any private information shared		
	AOR* (95% CI)			AOR* (95% CI)			AOR* (95% CI)			AOR* (95% CI)		
	Ghana	Guinea	Nigeria	Ghana	Guinea	Nigeria	Ghana	Guinea	Nigeria	Ghana	Guinea	Nigeria
Curtains during labour												
No	**2.5 (1.4 to 4.7**)	**0.2 (0.1 to 0.4**)	1.9 (0.9 to 3.9)	**6.7 (2.9 to 14.9**)	**0.04 (0.02 to 0.08**)	**6.5 (2.9 to 14.5**)	**5.9 (2.8 to 12.9**)	**0.04 (0.02 to 0.07**)	**2.7 (1.3 to 5.9**)	**3.8 (1.6 to 8.9**)	1.7 (0.7 to 4.2)	**4.9 (1.9 to 12.7**)
Yes	Ref	Ref	Ref	Ref	Ref	Ref	Ref	Ref	Ref	Ref	Ref	Ref

*Adjusted for women’s age, education and marital status.

AOR, adjusted OR; ref, reference.

## Discussion

This WHO multicountry study provides significant insight into the occurrence and determinants of different types of mistreatment of women during vaginal examination during childbirth across Ghana, Nigeria and Guinea using direct labour observations. Using a standardised labour observation tool, we ascertained how different types of mistreatment varied across countries. Across the three countries, women were consistently observed to experience non-consented care across any or multiple vaginal examinations, whereas for other types of mistreatment, it varied (private information shared, breast and genitalia exposure) across vaginal examinations. In particular, women in Nigeria experienced more accounts of each type of mistreatment during vaginal examinations, which may be due to the lack of curtains to ensure privacy of the woman’s body and some protection from disclosing personal information to others.

While there is a paucity of evidence on mistreatment during vaginal examinations, various studies have reported the prevalence of this phenomenon. A study in Pakistan used direct observations of labour and found that among women who received vaginal examination, 54% of examinations were performed without informed consent,^26^ similar to the 59% observed in our study. In terms of other types of mistreatment, studies have shown that women’s private information is shared during examinations (referred to as non-confidential care in other studies); however, it varies across studies that have used different methodologies: 54% in a study in Tanzania using community follow-up,^15^ 5.6% in a community-based study in India^27^ and 11.5% among postnatal women in Saudi Arabia.^28^ In our study, we also found substantial variation in women who had private information shared across the countries, ranging from <5% in Ghana to approximately 50% in Nigeria.

The association between experiences of different types of mistreatment across any vaginal examinations and absence of curtains during labour varied significantly by country. Overall, after adjusting for women’s sociodemographic characteristics, the absence of curtains during labour was associated with an increased risk of vaginal examination-related mistreatment during childbirth in Nigeria (more than fivefold increased risk of genital and breast exposure) and Ghana (more than twofold increased risk of private information shared), but a decreased risk of all vaginal examination-related mistreatment in Guinea. Our findings from the labour observations are consistent with the community survey data within the same WHO multicountry study, which showed that the association between some types of mistreatment (ie, physical abuse, verbal abuse, poor communication and the absence of a labour companion) also varied by country.^29^ Absence of a labour companion was associated with non-consented vaginal examination in Ghana and Guinea but not Nigeria, which only showed a significant association with decreased waiting time.^29^ In this study, non-consented vaginal examination was generally high across all the three countries, ranging from approximately 50% in Guinea to nearly 70% in Nigeria. The burden of non-consented vaginal examination seems widespread and under-reported. In a study among maternity care providers in Czech Republic, over 50% did not obtain women’s consent for vaginal examination at the facility level, while permission was always sought during home birth.^30^ Differences in the types of mistreatment during vaginal examination across countries highlight the importance of the role of context implications in the interpretation of our findings. While there is limited evidence on interventions for addressing mistreatment, health systems need context-specific adaptations to interventions, which may include improved privacy measures and training of health workers on improved communication, to improve women’s experiences of care.

Across the three countries, the different types of mistreatment women experience during vaginal examinations have implications for women’s general experience of care. First, evidence shows women are generally uncomfortable during a vaginal examination; in the community survey component of this study, approximately 30% of women reported feeling very uncomfortable during vaginal examinations (19.5% in Guinea, 37.4% in Nigeria and 45.9% in Ghana). While this may be due to the inherently invasive nature of vaginal examinations, more can be done to improve communication and consent before the exam and to improve the manner in which examinations are conducted. Simulated healthcare professional trainings to improve provider–women communication, informed consent care and ensuring adherence to women’s privacy measures should be emphasised such that women can be comfortable and experience better care during vaginal examinations.^31^ For instance, a recent pilot (baseline and endline) study in Ghana evaluated the impact of an integrated simulation-based training on provision of respective maternity care and reported significant improvement in the quality of care: about 15% in dignity and respect, 55% in supportive care and 87% in communication and autonomy.^32^ Relaxation methods may also minimise the discomfort and pain experienced by birthing women during vaginal examinations.^23^

The different types of mistreatment occurring during vaginal examinations are not aligned with quality of care standards and may in some cases violate women’s rights. The WHO Standards for Quality of Maternal and Newborn Care in Health Facilities states that all women should have informed choices in the care they receive, and the need for interventions should be clearly explained.^33^ The quality of care experienced by women remains an important driver to improve maternal and newborn health as increasing the coverage of essential interventions alone is not adequate to reduce maternal mortality and severe morbidity.^7 34^

### Strengths and limitations

The strengths of the study include use of large WHO multicountry data and standardised tool. In this study, we determined country-specific differences in the occurrence of mistreatment during vaginal examination. The use of direct observation of the labour events is considered a strength of the study as this allowed direct visualisation of the occurrence of mistreatment during vaginal examinations. Also, using non-health workers as direct observers of the labour events resulted in objective documentation of the events that occurred during the course of labour. This approach is considered superior to reports by the women themselves due to the potential risk of recall bias. While direct observations of care may positively influence health worker behaviour (Hawthorne effect), we explored this and found no evidence of its presence (by facility, country or month of recruitment).^13^

The limitations of our analysis include the fact that the timing of vaginal examination and the results of the assessment (eg, cervical dilation) were not explored in women who received multiple examinations. This could provide insight into the how a woman’s labour progressed in cases of mistreatment. In addition, other relevant information such as volume of deliveries (including episiotomy) and other types of mistreatment was reported in the main paper and not included in the analysis. Furthermore, we have limited data on other contextual facility-level (eg, supplies) and provider-level (eg, type of provider) factors as determinants of mistreatment during vaginal examination. We would also like to note that estimates of cervical dilation are often imprecise and can vary significantly both between examinations and across health workers. Also, non-communication of the vaginal examination findings with the women may constitute a significant component of mistreatment. However, this was beyond the scope of our study and was not reported. Despite these limitations, the findings of this study remain relevant in improving respective maternity care during childbirth in health facilities.

### Implications for research and practice

Our study showed that different types of mistreatment occur frequently during vaginal examinations across multiple countries. Regular training of maternal healthcare providers (eg, refresher courses, preservices and in-service) on the proper etiquette of conducting a woman-centred vaginal examination including optimal informed consent may help to enhance and promote positive childbirth experience.^3^ Given the sensitive nature of vaginal examination, the standard guidelines should be well integrated in the teaching curriculum and well taught in training institutions (eg, midwifery and medical schools). Furthermore, as part of routine antenatal care, education and counselling of pregnant women about experiencing vaginal examinations during labour, including why they are conducted, what they feel like, how often they might happen and what the risks are, may help women to feel more prepared for what to expect during childbirth. Further research is needed into the training standards to improve clinical skills in conducting respectful vaginal examinations (eg, consent, health literacy, communication and privacy) while reducing discomfort for women as much as possible.

Health systems must ensure enabling environments to support healthcare workers, including appropriate continuing education, supervision and supportive policies to promote respectful care.^7^ By adopting and implementing the WHO recommendations on intrapartum care and WHO standards for improving quality of maternal and newborn care in health facilities, health systems can achieve respectful maternity care for their users.^3 33^

## Conclusion

Our findings indicate different types of mistreatment of women (including non-consented care, lack of privacy and exposure of genitalia and breasts) are observed across any vaginal examinations and differently across the countries included in the study (Ghana, Nigeria and Guinea). The study strongly suggests the need to ensure better communication and consent processes prior to undertaking vaginal examination, in addition to the core principles of respective maternity care. Refresher courses for health providers and appropriate integration and teaching of the standard guidelines for performing vaginal examinations in training institutions are strongly recommended to ensure positive intrapartum care experience for birthing women. Furthermore, across different settings, health system interventions including availability of curtains can help increase women’s privacy during vaginal examinations. Different forms of mistreatment during vaginal examination have implications for women’s general experiences of care, quality of care standards and may even violate women’s rights. The country-level differences of mistreatment during vaginal examinations indicate context-specific interventions are necessary to scale up respectful maternity care globally.

## Data Availability

Data are available upon reasonable request. The analytic study dataset from the “WHO Study: How women are treated during facility-based childbirth” is de-identified and, archived through WHO/HRP’s electronic record management system. Data requests with an expression of interest in pursuing multi-country secondary analyses with a specific research question can be made to srhmph@who.int. More information about the study tools are available here:https://bmcmedresmethodol.biomedcentral.com/articles/10.1186/s12874-018-0603-x and the primary publication from the study here: https://www.thelancet.com/journals/lancet/article/PIIS0140-6736(19)31992-0/fulltext.
