# Supplementary material for: Vaginal examinations and mistreatment of women during facility-based childbirth in health facilities: secondary analysis of labour observations in Ghana, Guinea and Nigeria
Source: BMJ Glob Health. 2021 Nov 17;5(Suppl 2):e006640. doi: 10.1136/bmjgh-2021-006640 (PMC8733942; doi:10.1136/bmjgh-2021-006640)

Supplementary File

Annex 1. Different types of mistreatment across multiple vaginal examinations by country

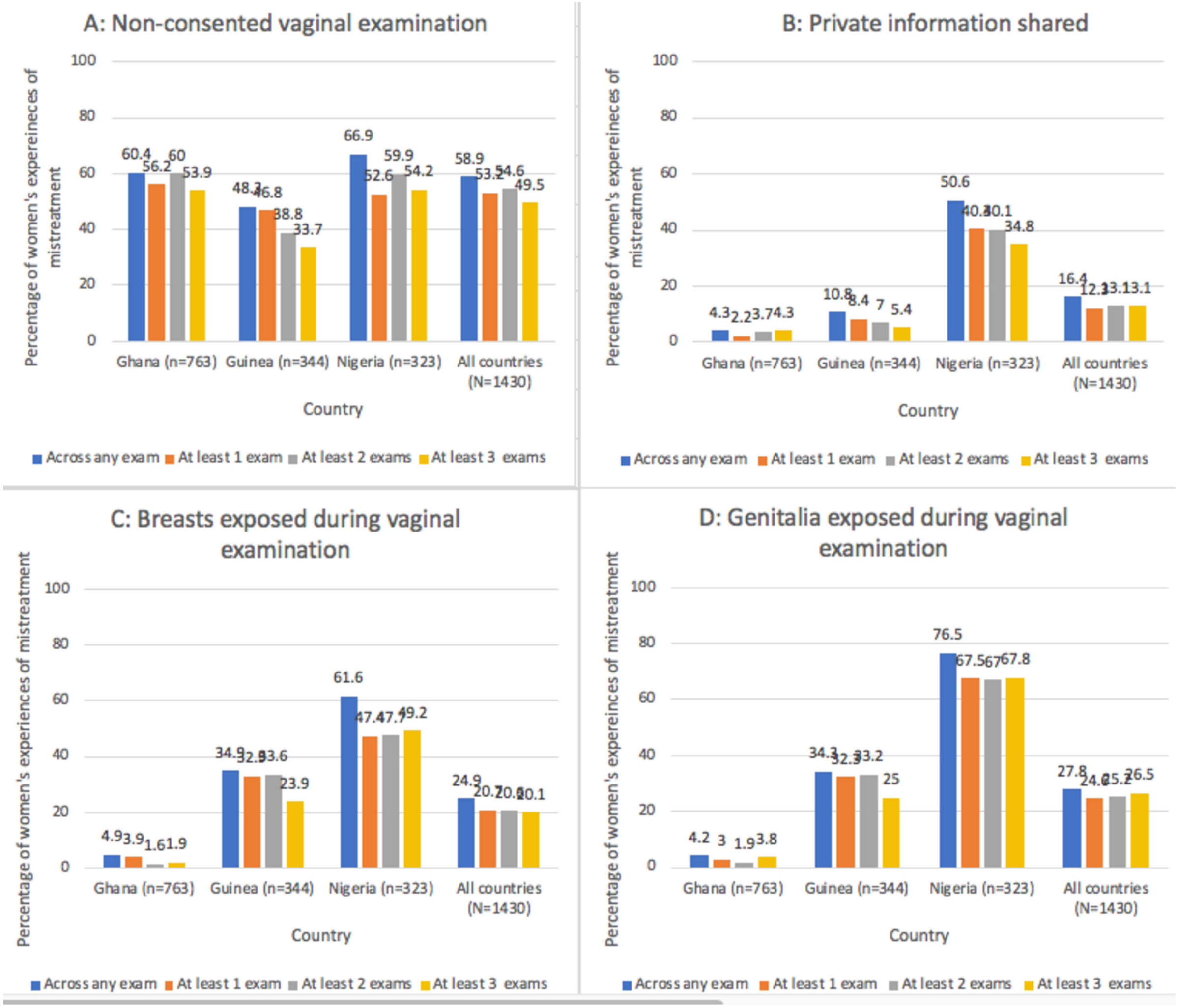

Supplement: Supplementary data [file bmjgh-2021-006640supp001.pdf]
